# Author Correction: Viral Bcl2s’ transmembrane domain interact with host Bcl2 proteins to control cellular apoptosis

**DOI:** 10.1038/s41467-021-22797-7

**Published:** 2021-04-08

**Authors:** Maria Jesús García-Murria, Gerard Duart, Brayan Grau, Elisabet Diaz-Beneitez, Dolores Rodríguez, Ismael Mingarro, Luis Martínez-Gil

**Affiliations:** 1 Department of Biochemistry and Molecular Biology, Institut de Biotecnologia i Biomedicina, Universitat de València, 46100 Burjassot, Spain; 2 Department of Molecular and Cell Biology, Centro Nacional de Biotecnología, Consejo Superior de Investigaciones Científicas, Campus Universidad Autónoma, 28049 Madrid, Spain

**Keywords:** Viral proteins, Virus-host interactions

Correction to: *Nature Communications* 10.1038/s41467-020-19881-9, published online 27 November 2020.

The original version of this Article contained an error in the Acknowledgements:

PROMETEOII/2014/061 should have read PROMETEO/2019/065

This has now been corrected in both the PDF and HTML versions of the Article.

