# Supplementary material for: Viral Bcl2s’ transmembrane domain interact with host Bcl2 proteins to control cellular apoptosis
Source: Nat Commun. 2020 Nov 27;11:6056. doi: 10.1038/s41467-020-19881-9 (PMC7695858; doi:10.1038/s41467-020-19881-9)
Supplement: Supplementary file 1 — Supplementary Information [file 41467_2020_19881_MOESM1_ESM.pdf]

Viral Bcl2s' transmembrane domain interact with host Bcl2 proteins to control cellular apoptosis

Supplementary Files

Maria Jesús García-Murria[1‡], Gerard Duart[1‡], Brayan Grau[1], Elisabet Diaz-Beneitez[2], Dolores Rodríguez[2], Ismael Mingarro[1], Luis Martínez-Gil[1*]

[1]Department of Biochemistry and Molecular Biology, Institut de Biotecnologia i Biomedicina, Universitat de València, 46100 Burjassot, Spain.

[2]Department of Molecular and Cell Biology, Centro Nacional de Biotecnología Consejo Superior de Investigaciones Científicas, Campus Universidad Autónoma, 28049 Madrid, Spain.

[‡]These authors contributed equally

[*]Author to whom correspondence might be addressed. Luis Martínez-Gil, Luis.martinez-gil@uv.es

TMD identified by the ΔG prediction server
TMD identified by the TMHMM Server v.2.0
TMD Incorporated in our assays

Herpesvirus

>G3CKQ0 **HHV4** Apoptosis regulator BHRF1
MAYSTREILLALCIRDSRVHGNGTLHPVLELAARETPLRLSPEDTVVLRYHVLLEEIIERNSETFTETWNRFITHTEHLD
LDFNSVFLEIFHRGDPSLGRALAWMAWCMHACRTLCCNQSTPYYVVDLSVRGMLEASEGLDGWIHQQGGWSTLIEDNIPG
SRR**FSWTLFLAGLTLSLLVICSYLFI**SRGRH
Predicted ΔG -2.632

>F5HGJ3 **HHV8** Apoptosis regulator Bcl-2 homolog
MDEDVLPGEVLAIEGIFMACGLNEPEYLYHPLLSPIKLYITGLMRDKESLFEAMLANVRFHSTTGIQLGLSMLQVSGDGN
MNWGRALAILTFGSFVAQKLSNEPHLRDFALAVLPVYAYEAIGPQWFRARGGWRGLKAYCTQVLTRRRG**RRMTALLGSIA**
LLATILAAVAMSRR
Predicted ΔG -1.008

>Q9WH78 **BoHV4** V-Bcl-2-like protein
MSLFFVVWYWVNYITKVCSGEVYIPSVLKFQYHSDTEHEPYSNLCKNLITMAEQDMDEVVSTIRRLLVECGMGLEEYLEH
PVTAPIKVAVQDVIRTKQDIFSNFLTNINSVEDLETLGHAITTLNDYPSPNMGRVVCGIAFSVYVVQTVCKRKPLLVRCC
LDIFTRATVQALNVNWFLQEGGWPALASFCKVVNSPSPRS**RWLFPMFAISGLVLTVGVAR**NMVHFT
Predicted ΔG -0.031

Poxvirus

>Q77PA8 **MyxV** Apoptosis regulator M11L
MMSRLKTAVYDYLNDVDITECTEMDLLCQLSNCCDFINETYAKNYDTLYDIMERDILSYNIVNIKNTLTFALRDASPSVK
LATLTLLASVIKKLNKIQHTDAAMFSEVIDGIVAEEQQVIGFIQKKCKYNTTYYNVRSGGCK**ISVYLTAAVVGFVAYGIL**
KWYRGT
Predicted ΔG -0.217

>P24356 **VacV** Protein F1L
MLSMFMCNNIVDYVDDIDNGIVQDIEDEASNNVDHDYVYPLPENMVYRFDKSTNILDYLSTERDHVMMAVRYYMSKQRLD
DLYRQLPTKTRSYIDIINIYCDKVSNDYNRDMNIMYDMASTKSFTVYDINNEVNTILMDNKGLGVRLATISFITELGRRC
MNPVETIKMFTLLSHTICDDYFVDYITDISPPDNTIPNTST**REYLKLIGITAIMFATYKTL**KYMIG
Predicted ΔG 0.902

>A0A0R8HV90 **OrfV** Apoptosis inhibitor
MANRDDIDASAVMAAYLAREYAEAVEEQLTPRERDALEALRVSGEEVRSPLLQELSNAGEHRANPENSHIPAALVSALLE
APTSPGRMVTAVELCAQMGRLWTRGRQLVDFMRLVYVLLDRLPPTADEDLGAWLQAVARVHGTRRRLH**RALGVGAVVAGV**
GMLLLGVRVLRRT
Predicted ΔG -0.815

Supplementary Figure 1. **vBcl2 sequences**. The figure includes the sequences (in a FASTA format) of the HHV4 BHRF1, HHV8 ORF16, BoHV4 ORF16, MyxV M11L, VacV F1L, and OrfV ORFV125 proteins. The Uniprot code is shown alongside the viral acronym and the protein name. The TMDs identified by the ΔG prediction server using the full protein scan mode are highlighted in yellow, the TMDs predicted by the TMHMM Server are underlined, and the hydrophobic sequences used in our assays are in bold. Included below each sequence is the predicted ΔG, calculated using the ΔG prediction server.

TMD Incorporated in our assays

>P10415 **Bcl2** Apoptosis regulator Bcl-2
MAHAGRTGYDNREIVMKYIHYKLSQRGYEWDAGDVGAAPPGAAPAPGIFSSQPGHTPHPAASRDPVARTSPLQTPAAPGA
AAGPALSPVPPVVHLTLRQAGDDFSRRYRRDFAEMSSQLHLTPFTARGRFATVVEELFRDGVNWGRIVAFFEFGGVMCVE
SVNREMSPLVDNIALWMTEYLNRHLHTWIQDNGGWDAFVELYGPSMRPLFDFS**WLSLKTLLSLALVGACITLGAYL**GHK

>Q07817 **BclXL** Bcl-2-like protein 1
MSQSNRELVVDFLSYKLSQKGYSWSQFSDVEENRTEAPEGTESEMETPSAINGNPSWHLADSPAVNGATGHSSSLDAREV
IPMAAVKQALREAGDEFELRYRRAFSDLTSQLHITPGTAYQSFEQVVNELFRDGVNWGRIVAFFSFGGALCVESVDKEMQ
VLVSRIAAWMATYLNDHLEPWIQENGGWDTFVELYGNNAAAESRKGQER**FNRWFLTGMTVAGVVLLGSLF***SR*K

>Q07812 **Bax** Apoptosis regulator BAX
MDGSGEQPRGGGPTSSEQIMKTGALLLQGFIQDRAGRMGGEAPELALDPVPQDASTKKLSECLKRIGDELDSNMELQRMI
AAVDTDSPREVFFRVAADMFSDGNFNWGRVVALFYFASKLVLKALCTKVPELIRTIMGWTLDFLRERLLGWIQDQGGWDG
LLSYFGTP**TWQTVTIFVAGVLTASLTIW**KKMG

>Q16611 **Bak** Bcl-2 homologous antagonist/killer
MASGQGPGPPRQECGEPALPSASEEQVAQDTEEVFRSYVFYRHQQEQEAEGVAAPADPEMVTLPLQPSSTMGQVGRQLAI
IGDDINRRYDSEFQTMLQHLQPTAENAYEYFTKIATSLFESGINWGRVVALLGFGYRLALHVYQHGLTGFLGQVTRFVVD
FMLHHCIARWIAQRGGWVAALNLGNGP**ILNVLVVLGVVLLGQFVVRRFF**KS

>Q96LC9 **Bmf** Bcl-2-modifying factor
MEPSQCVEELEDDVFQPEDGEPVTQPGSLLSADLFAQSLLDCPLSRLQLFPLTHCCGPGLRPTSQEDKATQTLSPASPSQ
GVMLPCGVTEEPQRLFYGNAGYRLPLPASFPAVLPIGEQPPEGQWQHQAEVQIARKLQCIADQFHRLHVQQHQQ**NQNRVW**
WQILLFLHNLALNGEENRNGAGPR

>Q13323 **Bik** Bcl-2-interacting killer
MSEVRPLSRDILMETLLYEQLLEPPTMEVLGMTDSEEDLDPMEDFDSLECMEGSDALALRLACIGDEMDVSLRAPRLAQL
SEVAMHSLGLAFIYDQTEDIRDVLRSFMDGFTTLKENIMRFWRSPNPGSWVSCEQV**LLALLLLLALLLPLLSGGLHLLL**K

>Q13794 **Noxa** Phorbol-12-myristate-13-acetate-induced protein 1
MPGKKARKNAQPSPARAPAELEVECATQLRRFGDKLNFRQKLLNLISKLFCSGT

>Q15388 **Tomm20** Mitochondrial import receptor subunit TOM20 (Human)
MVGRNS**AIAAGVCGALFIGYCIY**FDRKRRSDPNFKNRLRERRKKQKLAKERAGLSKLPDLKDAEAVQKFFLEEIQLGEEL
LAQGEYEKGVDHLTNAIAVCGQPQQLLQVLQQTLPPPVFQMLLTKLPTISQRIVSAQSLAEDDVE
Predicted ΔG 0.217

>Q9NS69 **Tomm22** Mitochondrial import receptor subunit TOM22 (Human)
MAAAVAAAGAGEPQSPDELLPKGDAEKPEEELEEDDDEELDETLSERLWGLTEMFPERVRSAAGATFDLSLFVAQKMYRF
SRA**ALWIGTTSFMILVLPVVFET**EKLQMEQQQQLQQRQILLGPNTGLSGGMPGALPSLPGKI

>P00803 **Lep** Leader Signal peptidase I (E.coli)
MANMFALILVIATLVTGILWCVDKFFFAPKRRERQAAAQAAAGDSLDKATLKKVAPKPG**WLETGASVFPVLAIVLIVRSF**
IYEPFQIPSGSMMPTLLIGDFILVEKFAYGIKDPIYQKTLIETGHPKRGDIVVFKYPEDPKLDYIKRAVGLPGDKVTYDP
VSKELTIQPGCSSGQACENALPVTYSNVEPSDFVQTFSRRNGGEATSGFFEVPKNETKENGIRLSERKETLGDVTHRILT
VPIAQDQVGMYYQQPGQQLATWIVPPGQYFMMGDNRDNSADSRYWGFVPEANLVGRATAIWMSFDKQEGEWPTGLRLSRI
GGIH

>P02724 **GpA** Glycophorin-A (Human)
MYGKIIFVLLLSEIVSISASSTTGVAMHTSTSSSVTKSYISSQTNDTHKRDTYAATPRAHEVSEISVRTVYPPEEETGER
VQLAHHFSEPE**ITLIIFGVMAGVIGTILLISYGI**RRLIKKSPSDVKPLPSPDTDVPLSSVEIENPETSDQ

Supplementary Figure 2. **cBcl2s and control protein sequences. a,** The panel includes the sequences (in a FASTA format) of Bcl2, BclXL, Bax, Bak, Bik, and Bmf. The TMD in each protein is shown in bold. **b,** The panel includes the sequences of the control proteins used in this manuscript, Tomm20 (T20), Tomm22 (T22), Lep and GpA. The TMDs are shown in bold. In those cases where the protein has two TMDs, only the one used as control is marked. The Uniprot code of each protein is shown alongside its name and the species to which it belongs.

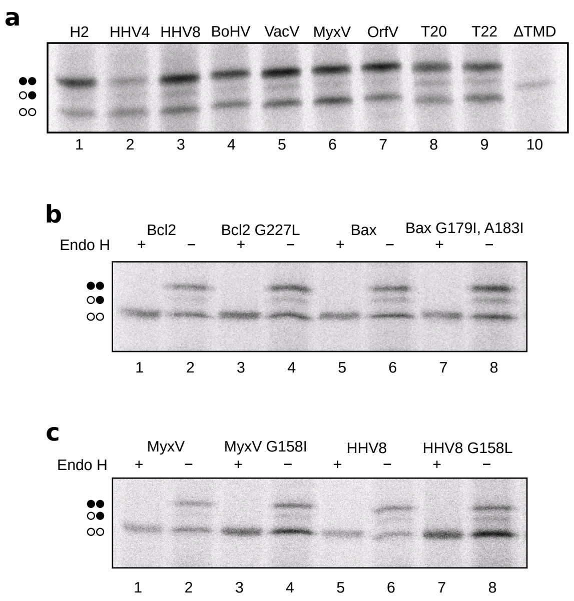

Supplementary Figure 3. **Insertion of vBcl2 Ct hydrophobic sequences in ER-derived microsomes**. **a**, In vitro translation of Lep variants where H2 has been replaced by the Ct hydrophobic region of the indicated vBcl2 proteins. As a translocation control, we used a Lep variant where H2 has been removed (ΔTMD), ΔGexp=1.50 kcal/mol. Absence of glycosylation of G1 and G2 acceptor sites is indicated by two white dots, single glycosylation by one white and one black dot, and double glycosylation by two black dots (n=3). **b** and **c**, In vitro protein translation in the presence of ER-derived microsomes. After translation Lep chimeras bearing the TMD of Bcl2, Bcl2 G227L, Bax and Bax G179I, A183I, MyxV, MyxV G158I, HHV8, or HHV G158L were treated with (+) or without (-) of Endoglycosydase H (EndoH) (n=3).

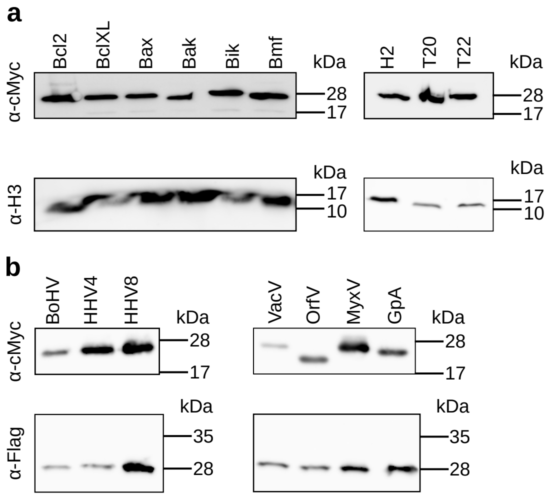

Supplementary Figure 4. **BiFC WB quantification**. **a**, WB analysis of VN chimeras bearing the TMD of Bcl2, BclXL, Bax, Bak, Bik, Bmf, H2, T20 or T22 using an anti-c-Myc antibody. Histone 3 (H3) was used as a loading control (n=3). **b**, WB quantification of VN chimeras bearing the TMD of BoHV, HHV4, HHV8, VacV, OrfV, MyxV, or GpA using an anti-c-Myc antibody (n=3). Cells were co-transfected with red fluorescent protein bearing a flag tag to facilitate protein loading quantification.

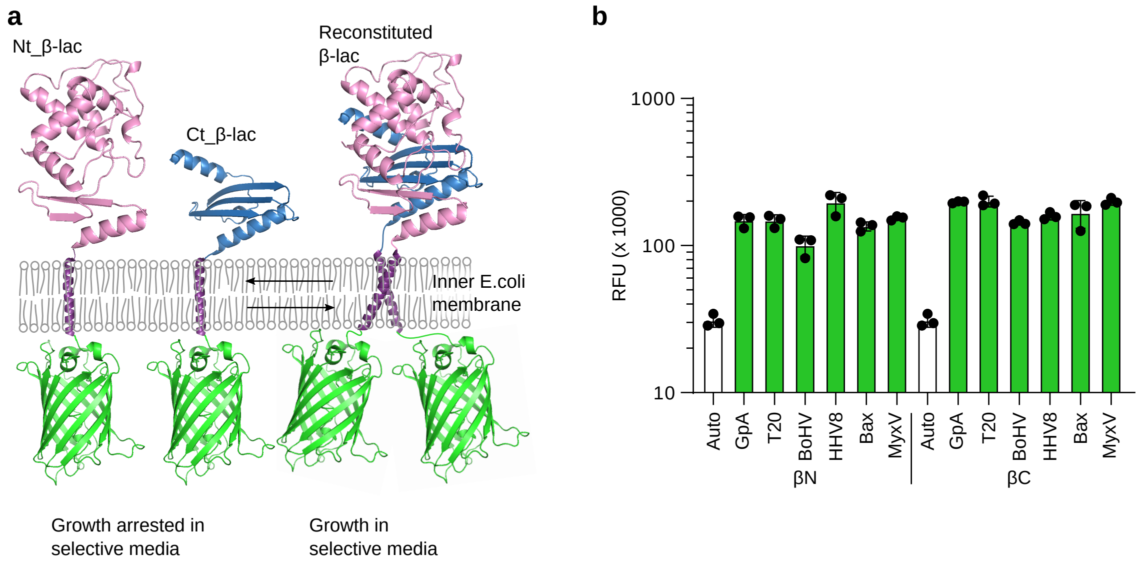

Supplementary Figure 5. **BlaTM assay in E. coli membranes**. **a**, A schematic representation of the BlaTM assay. The position of the Nt and Ct ends of the β-lactamase (in pink and blue respectively), the TMD (in purple), and the GFP (in green) are shown. βN and βC fragments (including the indicated TMDs) were expressed in E.coli in the presence of ampicillin at increasing concentrations. After 16 hours, the OD544 of the culture was measured and normalized based on the GpA homo-dimer values. The strength of the TMDs interaction is proportional to the LD50 of the antibiotic. **b**, Expression profile of all the BlaTM chimeras used in this work. To ensure that differences in the protein levels were not the source of the observed variations in LD50, we measured the GFP-derived fluorescence of all BlaTM chimeras. The mean and standard deviation fluorescence (Relative Fluorescence Units, RFU) of three independent experiments are shown (green bars) (n=3). Individual values are represented with solid dots.

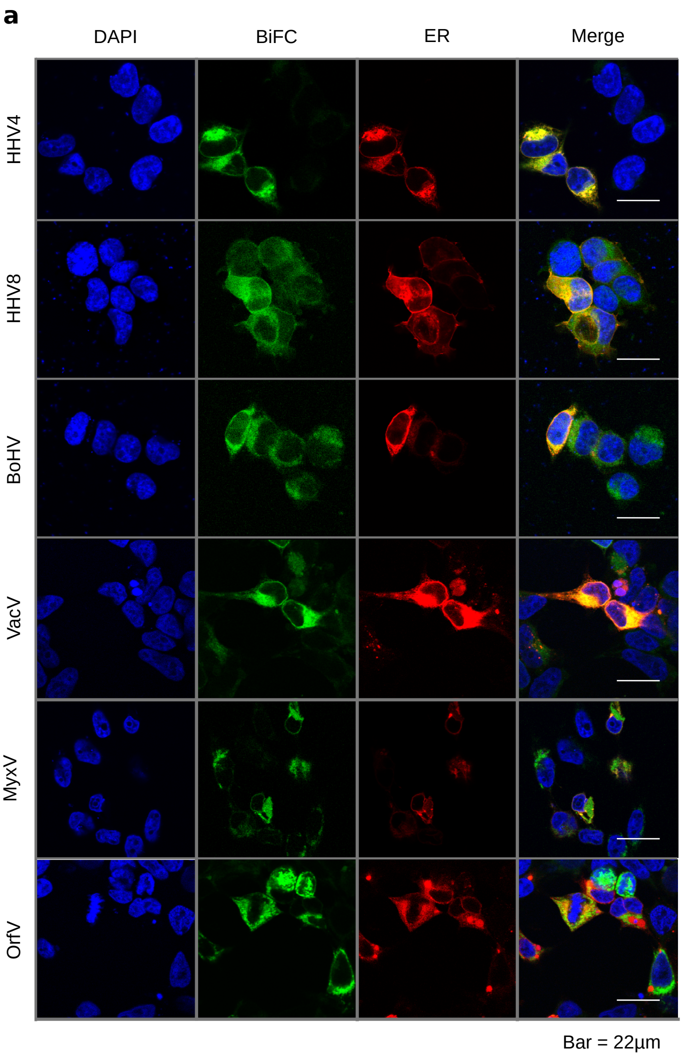

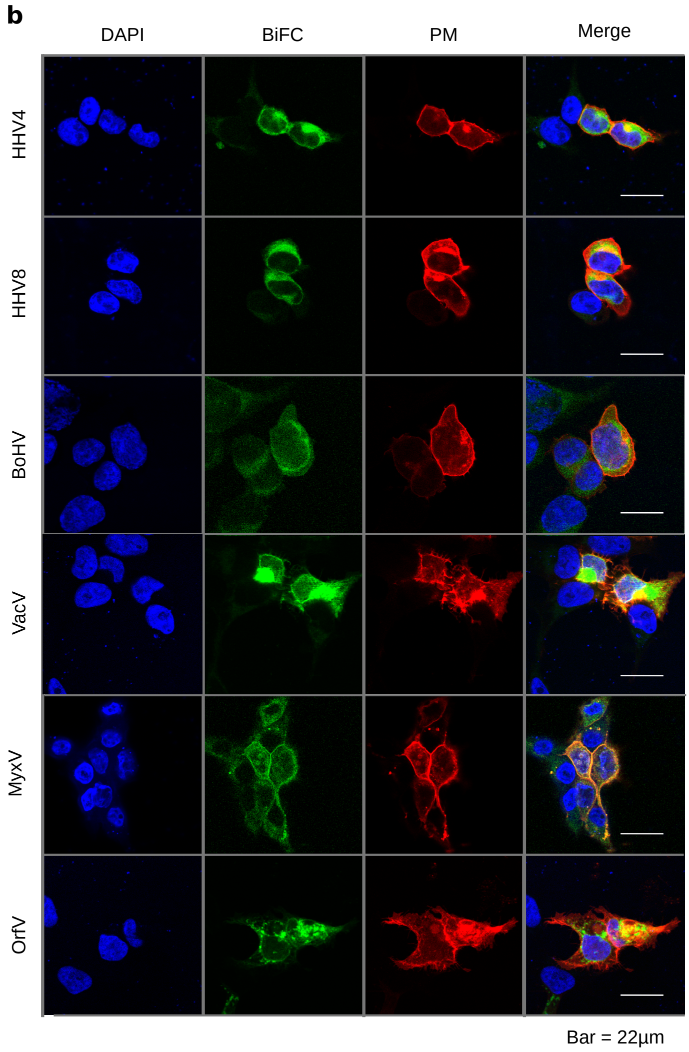

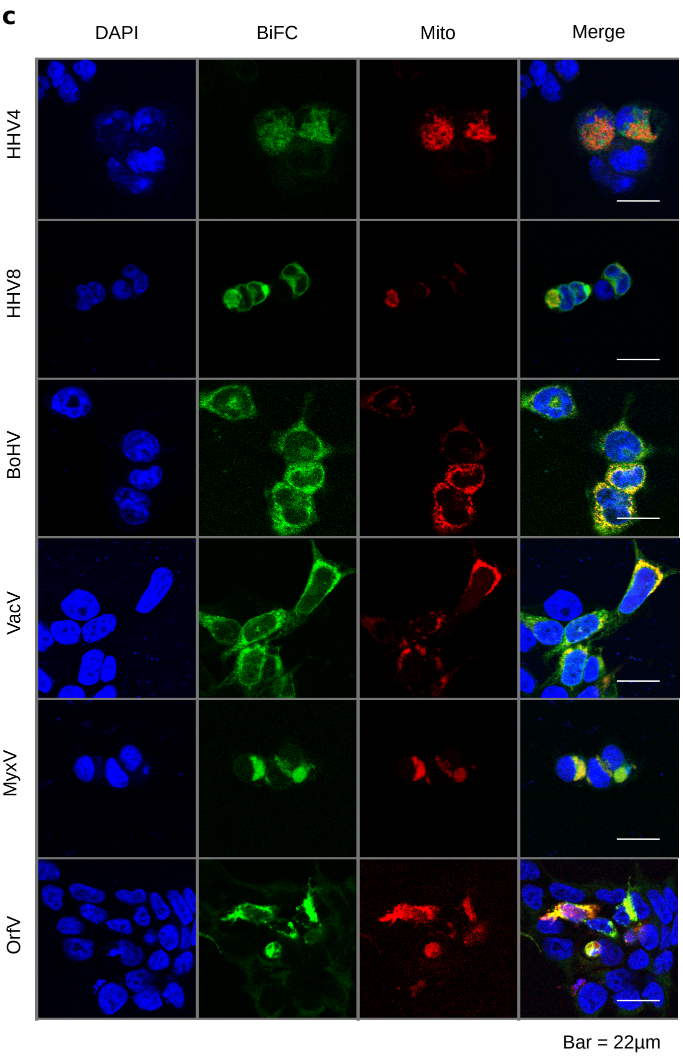

Supplementary Figure 6. **Localization of TMD homo-oligomers**. **a**, **b**, and **c**, The localization of TMD-TMD homo-oligomers was analyzed by fluorescent confocal microscopy (n=4). DAPI staining is shown in blue, the BIFC signal for each of the homo-oligomers in green, and the cellular marker in red (Endoplasmatic reticulum (ER), Plasma membrane (PM), Mitochondria (Mito)). The right column of each panel shows the co-localization of BiFC and the corresponding cellular marker in yellow (visible only when the images are merged).

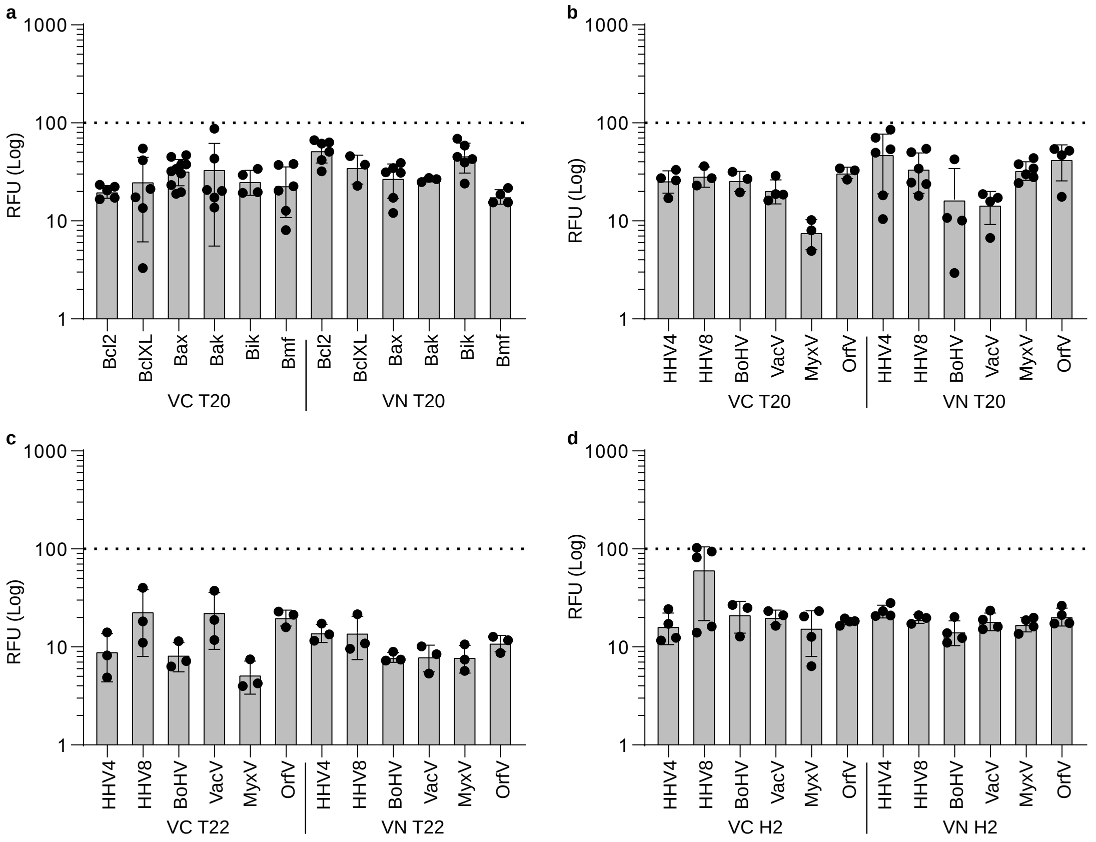

Supplementary Figure 7. **Hetero-oligomerization of vBcl2s TMDs and control sequences**. **a**-**d**, Relative fluorescence for the oligomerization of T20 TMD with vBcl2 or cBcl2 TMDs. c and d, Hetero-oligomerization of vBcl2s TMDs with T22 or Lep H2 TMDs. The bars show the mean and standard deviation of at least three independent experiments (n>3). The TMD included in the VFP chimeras (VN or VC) is indicated below each bar. Solid dots represent the results of individual experiments. The GpA TMD homo-dimer was used as a positive control and as the normalization value (dotted line). As negative controls, we used the interaction of each partner in the hetero-oligomers with T20 TMD (i.e., X/T20 and T20/Y for the X/Y interaction). An interaction was considered only if the fluorescence values were significantly higher (two-tailed homoscedastic t-test) than those of the two negative controls. No statistical differences were found.

**Virus**	**Host**	**Host scientific**
HHV4	Human	Homo sapiens
HHV8	Human	Homo sapiens
BoHV	Bovine	Bos taurus
VacV	Bovine/Horse	Bos taurus/Equus caballus
MyxV	Rabbit	Oryctolagus cuniculus
OrfV	Sheep/Goat	Ovis aries/Capra hircus

Bcl2

```
>Homo sapiens
WLSLKTLLSLALVGACITLGAYL
>Bos taurus
WLSLKALLSLALVGACITLGAYL
>Equus caballus
WLSLKALLSLALVGACITLGAYL
>Oryctolagus cuniculus
WVSLKTLFSLALIGACITLGAYL
>Capra hircus
WLSLKALLSLALVGACITLGAYL
```

```
Oryctolagus      WVSLKTLFSLALIGACITLGAYL
Homo             WLSLKTLLSLALVGACITLGAYL
Bos              WLSLKALLSLALVGACITLGAYL
Equus            WLSLKALLSLALVGACITLGAYL
Capra            WLSLKALLSLALVGACITLGAYL
                 *:***:*:****:**********
```

BclXL

```
>Homo sapiens
FNRWFLTGMTVAGVVLLGSLF
>Bos taurus
FNRWFLTGMTVAGVVLLGSLF
>Equus przewalskii†
FNRWFLTGMTVAGVVLLGSLF
>Oryctolagus cuniculus
FNRWFLTGMTVAGVVLLGSLF
>Ovis aries
FNRWFLTGMTVAGVVLLGSLF
```

```
Homo             FNRWFLTGMTVAGVVLLGSLF
Bos              FNRWFLTGMTVAGVVLLGSLF
Equus            FNRWFLTGMTVAGVVLLGSLF
Oryctolagus      FNRWFLTGMTVAGVVLLGSLF
Ovis             FNRWFLTGMTVAGVVLLGSLF
                 *********************
```

† The sequence from Equus przewalskii was used as a substitute of Equus caballus

Bax

```
>Homo sapiens
TWQTVTIFVAGVLTASLTIW
>Bos taurus
TWQTVTIFVAGVLTASLTIW
>Equus caballus
TWQTVTIFVAGVLTASLTIW
>Oryctolagus cuniculus
TWQTLTILGAGVLTASLTIW
>Capra hircus
TWQTVTIFVAGVLTASLTIW
>Ovis aries
TWQTVTIFVAGVLTASLTIW
```

```
Homo             TWQTVTIFVAGVLTASLTIW
Bos              TWQTVTIFVAGVLTASLTIW
```

```
Equus            TWQTVTIFVAGVLTASLTIW
Capra            TWQTVTIFVAGVLTASLTIW
Ovis             TWQTVTIFVAGVLTASLTIW
Oryctolagus      TWQTLTILGAGVLTASLTIW
                 ****:**: ***********
```

Bak

```
>Homo sapiens
ILNVLVVLGVVLLGQFVVRRFF
>Bos taurus
IKSVAIVLAVVLLGQFVVRRFF
>Equus caballus
IRNVLIVLAVVLLGQYVVRRFF
>Oryctolagus cuniculus
ILTVLAALAVVAFCQFVVRR
>Capra hircus
IKNVAIVLAVVLLGQFVVRRFF
>Ovis aries
IKNVAIVLAVVLLGQFVVRRFF
```

```
Oryctolagus      ILTVLAALAVVAFCQFVVRR--
Homo             ILNVLVVLGVVLLGQFVVRRFF
Equus            IRNVLIVLAVVLLGQYVVRRFF
Bos              IKSVAIVLAVVLLGQFVVRRFF
Capra            IKNVAIVLAVVLLGQFVVRRFF
Ovis             IKNVAIVLAVVLLGQFVVRRFF
                 * .*  .*.** : *:****
```

Bik

```
>Homo sapiens
LLALLLLLALLLPLLSGGLHLLL
>Equus caballus
LALSVLLLLVLLLGWGLHLL
```

```
Homo       LLALLLLLALLLPLLSGGLHLLL
Equus      -LALS-VLLLLVLLLGWGLHLL-
            ***  :* **: **. *****
```

Bmf

```
>Homo sapiens
NQNRVWWQILLFLHNLALNG
>Bos taurus
NRNRMWWQILLFLHNVALNG
>Oryctolagus cuniculus
NRNRVWWQILLFLHNLALNG
>Capra hircus
NRNRMWWQILLFLHNVALNG
>Ovis aries
NRNRMWWQILLFLHNVALNG
```

```
Bos             NRNRMWWQILLFLHNVALNG
Capra           NRNRMWWQILLFLHNVALNG
Ovis            NRNRMWWQILLFLHNVALNG
Homo            NQNRVWWQILLFLHNLALNG
Oryctolagus     NRNRVWWQILLFLHNLALNG
                *:**:**********:****
```

Supplementary Figure 8. **Alignment of cBcl2s TMDs.** The TMD sequence alignment for Bcl2, BclXL, Bax, Bak, Bik and, Bmf. The input sequences (in FASTA format) and the results of each alignment are shown. Additionally, the host affected by each of the viruses is indicated. The sequences of the human proteins were used as a reference. Sequences were obtained from the Uniprot and NCBI databases (December 2019). The alignment was done with Clustal Omega (EMBL-EBI).

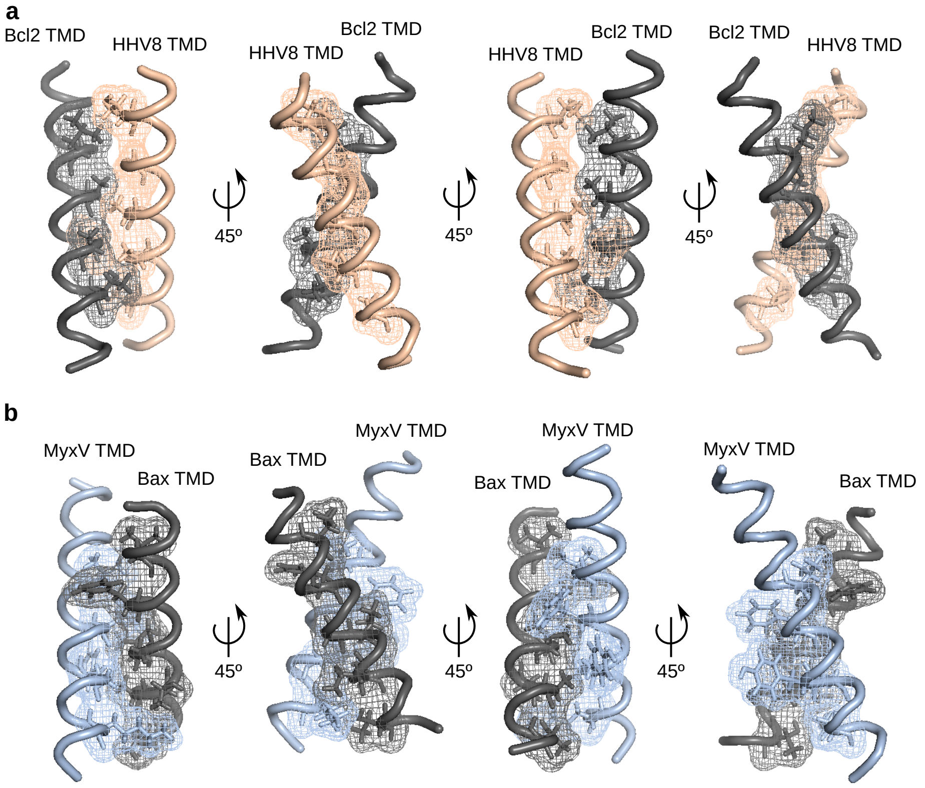

Supplementary Figure 9. **Models for the Bcl2-HHV8 and Bax-MyxV intramembrane interactions**. **a**, Model of a putative dimer between Bcl2 and HHV8 TMDs, obtained with PredDIMER. **b**, Model of a putative dimer between Bcl2 and MyxV TMDs, obtained with PredDIMER.

VN

VC	Bcl2	BclXL	Bax	Bak	Bik	Bmf	HHV4	HHV8	BoHV	VacV	MyxV	OrfV	T20	T22	H2
Bcl2	8E-06	n.d.	n.d.	n.d.	n.d.	n.d.	1E-05	0.0297	0.0039	0.0055	0.0005	0.0185	na	n.d.	n.d.
BclXL	n.d.	n.d.	n.d.	n.d.	n.d.	n.d.	0.929	0.229	0.810	0.0166	0.394	0.0445	na	n.d.	n.d.
Bax	n.d.	n.d.	n.d.	n.d.	n.d.	n.d.	0.879	0.672	0.557	0.0029	0.339	0.0124	na	n.d.	n.d.
Bak	n.d.	n.d.	n.d.	n.d.	n.d.	n.d.	0.388	0.486	0.315	0.0168	0.0113	0.0305	na	n.d.	n.d.
Bik	n.d.	n.d.	n.d.	n.d.	n.d.	n.d.	**0.0164**	0.514	0.654	0.0015	0.854	0.0397	na	n.d.	n.d.
Bmf	n.d.	n.d.	n.d.	n.d.	n.d.	n.d.	0.734	0.401	0.074	0.217	0.739	0.877	na	n.d.	n.d.
HHV4	0.0368	0.0403	0.418	0.626	0.0205	0.841	0.001	n.d.	n.d.	n.d.	n.d.	n.d.	na	0.136	0.237
HHV8	0.0009	0.0184	0.162	0.691	0.0003	0.0316	n.d.	0.0386	n.d.	n.d.	n.d.	n.d.	na	0.189	0.921
BoHV	0.179	0.753	0.626	0.837	0.416	0.151	n.d.	n.d.	0.1423	n.d.	n.d.	n.d.	na	0.444	0.817
VacV	0.005	0.0244	0.814	0.0164	0.0019	0.884	n.d.	n.d.	n.d.	0.0082	n.d.	n.d.	na	0.109	0.960
MyxV	0.0105	0.664	0.0367	0.0475	0.334	0.664	n.d.	n.d.	n.d.	n.d.	0.0028	n.d.	na	0.024	0.686
OrfV	0.0142	0.0222	0.501	0.597	0.497	0.536	n.d.	n.d.	n.d.	n.d.	n.d.	0.0002	na	0.053	0.778
T20	na	na	na	na	na	na	na	na	na	na	na	na	0.810	na	na
T22	n.d.	n.d.	n.d.	n.d.	n.d.	n.d.	0.138	0.682	0.123	0.781	0.261	0.465	na	0.394	n.d.
H2	n.d.	n.d.	n.d.	n.d.	n.d.	n.d.	0.801	0.251	0.658	0.932	0.748	0.714	na	n.d.	6E-07

Supplementary Figure 10. **Analysis of viral-host TMD-TMD interactions by BiFC**. The result of all the TMD-TMD interactions assayed in HEK 293T cells using the BiFC assay summarized in a matrix format. Fluorescence values (RFU) for each of the indicated BiFC combinations significantly higher than those of their respective negative controls are highlighted in green (two-tailed homoscedastic t-test, p-value < 0.05). Only the highest of the two corresponding p-values is shown. Absence of interaction is shown in white. Grey boxes indicate combinations not done (nd) or not applicable (na).

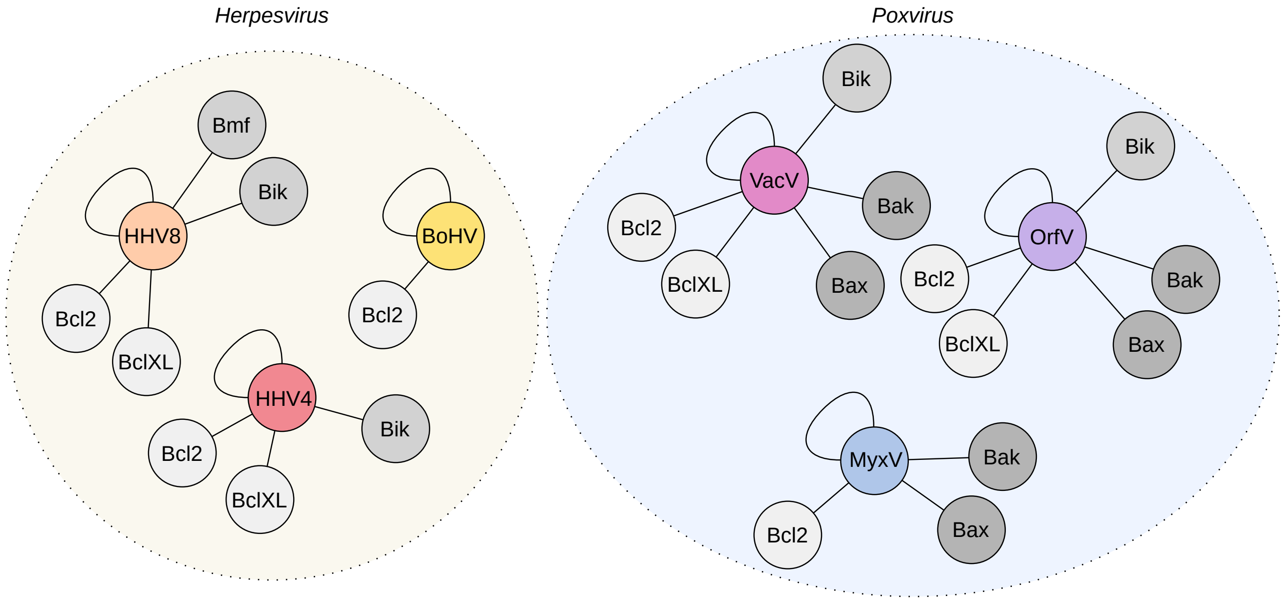

Supplementary Figure 11. **TMD-TMD interactions network of the vBcl2**. A network representation of the host-cell interaction of vBcl2 TMDs. The figure includes the results of the BiFC and BlaTM assays. Solid lines represent interactions, while TMDs are represented by nodes. The colored areas highlight the viral families, with poxviruses in light blue and herpesviruses in light yellow.

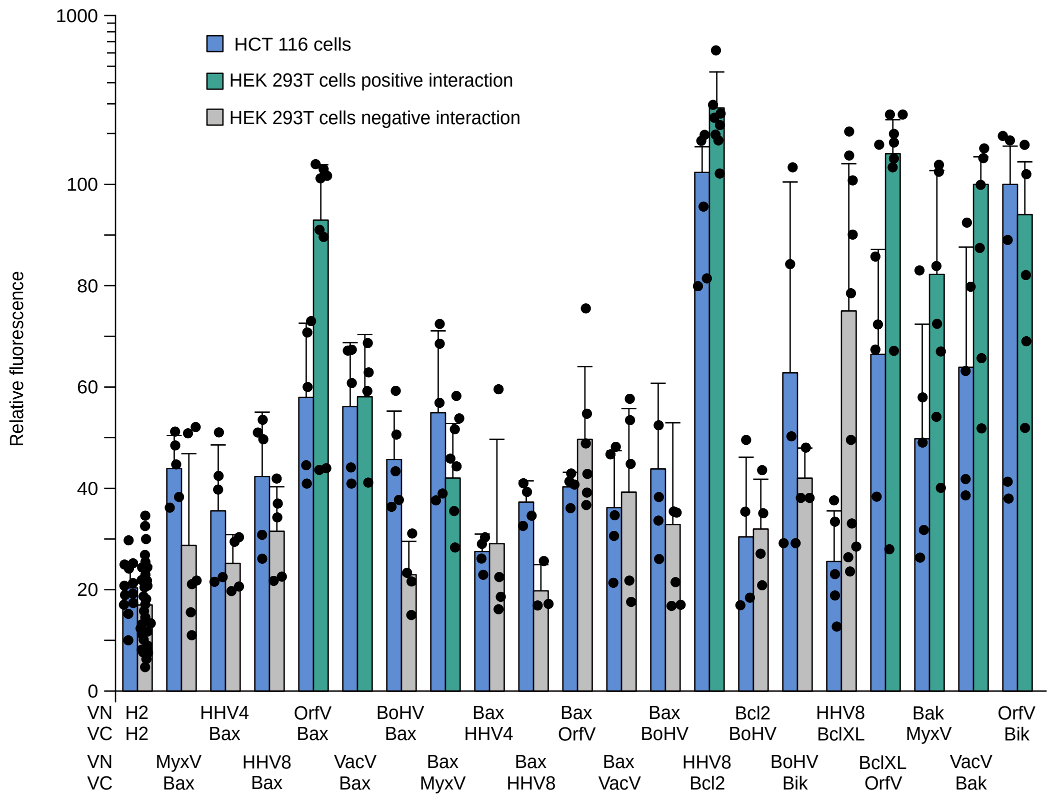

Supplementary Figure 12. **BiFC assay in HCT 116 cells**. Bar graph showing the relative fluorescence of the tested homo-oligomers in the BiFC assay in HCT 116 cells (blue bars). The mean and standard deviation of at least five independent experiments are shown (n>5). The corresponding value in HEK 293T cells is shown to facilitate comparison. In HEK 293T cells, those interactions that were statistically above their controls are shown in green, while grey bars denote the absence of intramembrane interaction (see Fig. 3).

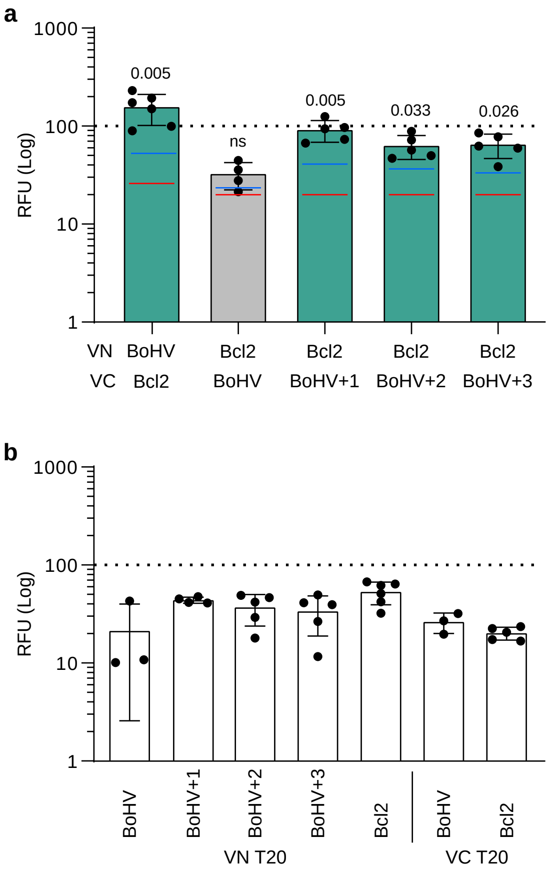

Supplementary Figure 13. **Analysis of the interaction between Bcl2 and BoHV TMDs**. **a** and **b**, Relative fluorescence (RFU) for the hetero-oligomerization between VN-Bcl2 and VC-BoHV, VC-BoHV+1, VC-BoHV+2, or VC-BoHV+3 (a). The results for the VN-BoHV/VC-Bcl2 oligomerization are included for comparison (previously shown in Fig 3.). The mean and standard deviation of at least three independent experiments are shown (n>3). Solid dots represent the results of individual experiments. The TMD included in the VFP chimeras (VN or VC) is indicated below each bar. The GpA TMD homo-dimer was used as a positive control and as the normalization value (dotted line). The interactions of each partner in the hetero-oligomers with T20 TMD were used as a negative control (b). To facilitate comparison, blue (VN/T20 TMD) and red (VC/T20 TMD) lines within each bar in panel a indicate the fluorescence of the corresponding controls. An interaction (highlighted in green) would be considered only if the corresponding fluorescence is significantly higher than those of the negative controls (two-tailed homoscedastic t-test). The highest of the two p-values is shown, ns non significant.

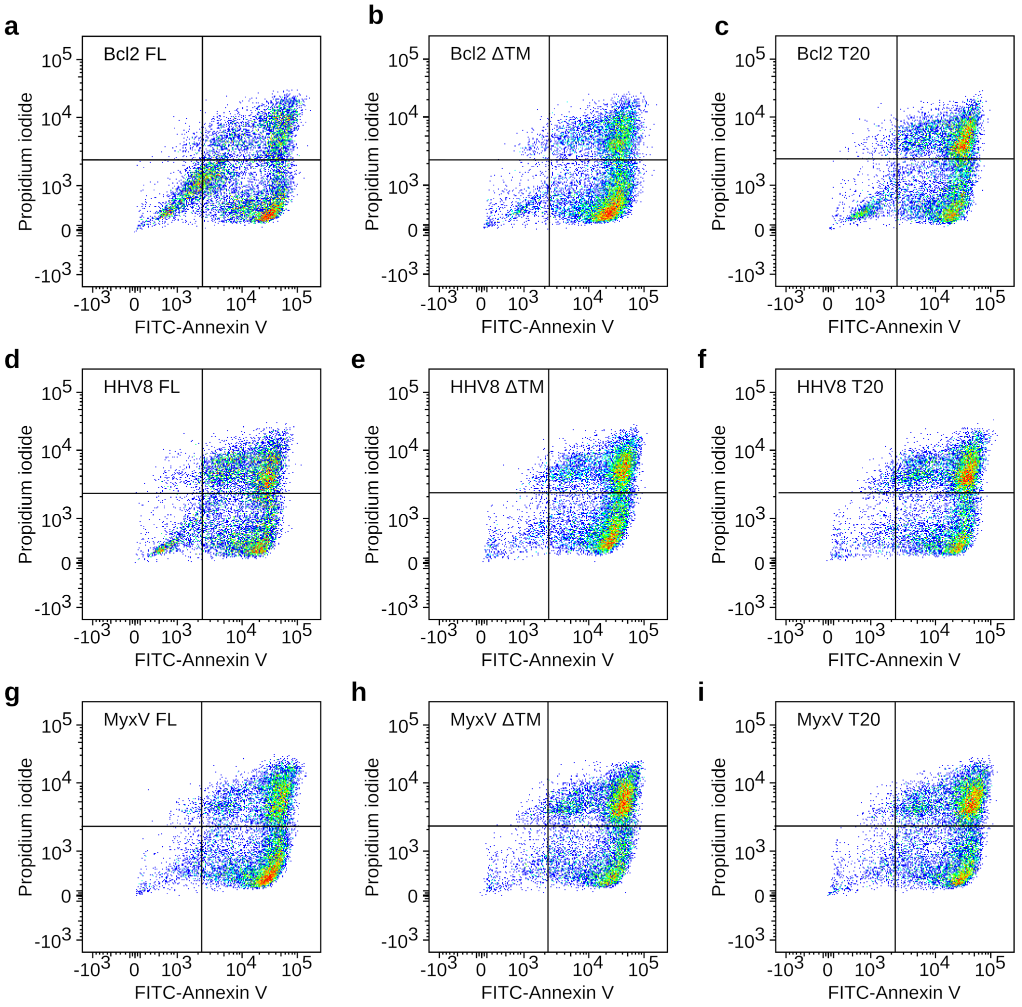

Supplementary Figure 14. **Flow cytometry analysis of vBcl2 TMD in apoptotic control**. **a-i**. Cells were transfected with Bcl2, HHV8 and MyxV with (FL) or without the TMD (ΔTMD), or with the TMD substituted by the TMD of T20 (T20), and treated with doxorubicin as an apoptotic stimulus. The percentage of cells in healthy, early apoptotic, late apoptotic, or necrotic states was measured by flow cytometry using propidium iody staining and phosphatidil serine labeling (FITC-Annexin V). Panels a-i show a representative plot for each of the indicated treatments.

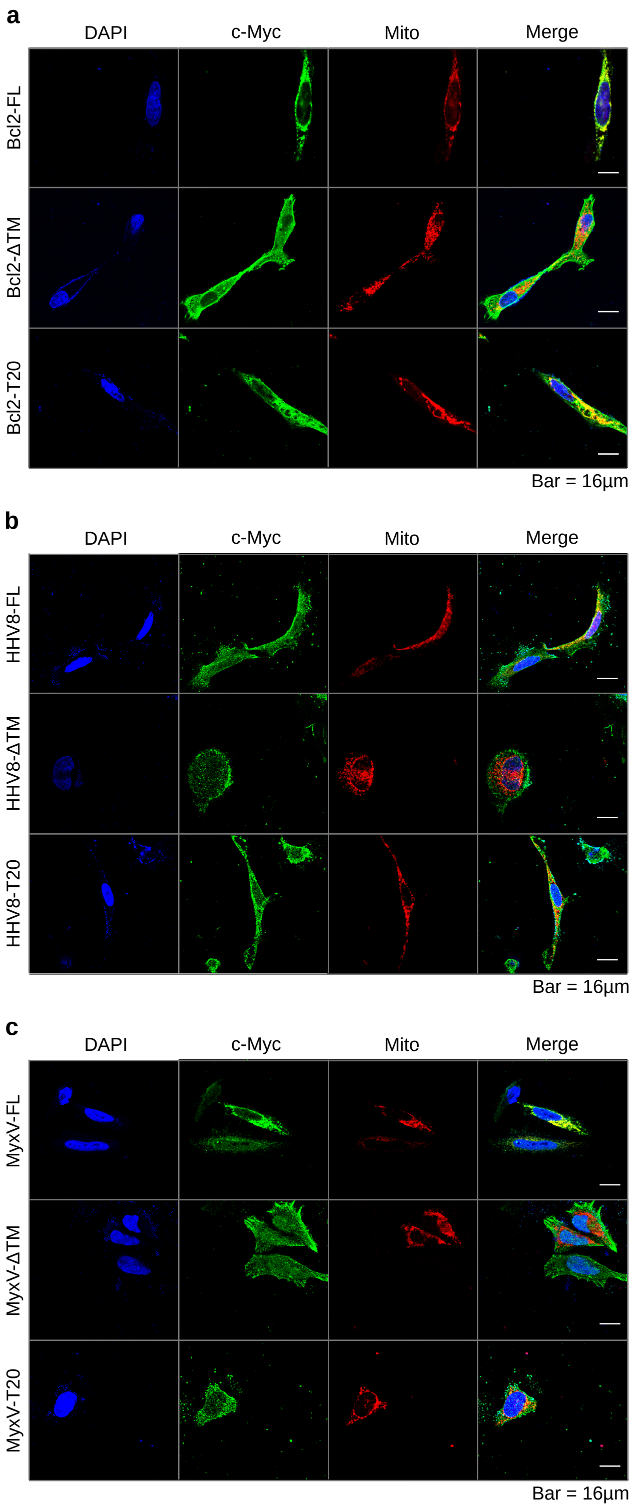

Supplementary Figure 15. **Sub-cellular localization of Bcl2, HHV8 and, MyxV**. **a-c**, Sub-cellular localization of Bcl2, HHV8 and MyxV with (FL) or without (ΔTMD) the TMD, or with the TMD replaced by the TMD of T20. vBcl2 proteins were transfected in HeLa cells and immunostained using an anti-c-Myc antibody (green) (n=4). To monitor mitochondrial localization, a marker (mCherry-T20) was co-transfected (red). DAPI staining is shown in blue. The right column of each panel shows the co-localization of the vBcl2 protein and the mitochondrial marker in yellow (visible only when the images are merged).

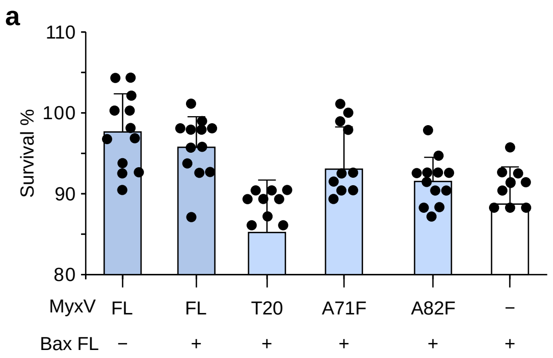

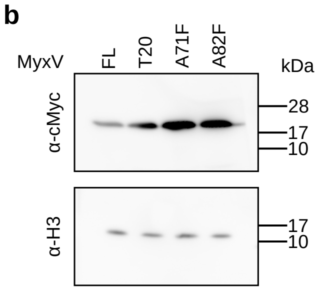

Supplementary Figure 16. **Influence of MyxV binding groove and TMD interactions on apoptotic control**. **a**, Cells were transfected with an empty plasmid (-), MyxV FL and T20, or MyxV bearing substitutions in alanines 71 or 82 to phenylalanines (A71F and A82F, respectively) and co-transfected with (+) or without (-) Bax FL as an apoptosis stimulus. Survival percentage mean and standard deviation of six replicates from two independent experiments are shown. **b**. Western blot analysis of protein levels. Histone 3 (H3) was used as a loading control (n=3).

Supplementary Table 1. **Used primers**

**Name**	**sequence**
vBcl2 gene string1_F	AATGTCACCCTAAGCTTCTTCGTGA
vBcl2 gene string1_R	AGAAGAAACCAACCTCGAGTCGGTA
vBcl2 gene string2_F	GTACAATGTCACCCTAGATCTTTTG
vBcl2 gene string2_R	TGTGAAGAAGAAACCAACGGTACCT
vBcl2 gene string3_F	TACAATGTCACCCTAAGCTTACCTG
vBcl2 gene string3_R	TGAAGAAGAAACCAACGGTGCCTCT
HHV4_Lep_F	GCATACTAGTTTTAGCTGGACTTTG
HHV4_Lep_R	GCATGGTACCCCATATAACTAACAG
HHV8_Lep_F	GCATACTAGTAGAATGACAGCGCTA
HHV8_Lep_R	GCATGGTACCCATCGCGACCGCTGC
BoHV4_Lep_F	GCATACTAGTCGCTCCAGATGGTTA
BoHV4_Lep_R	GCATGGTACCTCTCGCCACACCCAC
VacV_Lep_F	GCATACTAGTCGCGAGTACCTGAAA
VacV_Lep_R	GCATGGTACCCAGGGTTTTGTAGGT
MyxV_Lep_F	GCATACTAGTATCAGCGTGTACCTG
MyxV_Lep_R	GCATGGTACCTCTGTACCACTTCAG
OrfV_Lep_F	GCATACTAGTCGCGCCCTGGGCGTG
OrfV_Lep_R	GCATGGTACCGCGCAGCACGCGCAC
Bcl2_Lep_F	GCATACTAGTTGGCTGTCTCTGAAG
Bcl2_Lep_R	GCATGGTACCCAGATAGGCACCCAG
Bax_Lep_R	GCATACTAGTACCTGGCAGACCGTG
Bax_Lep_F	GCATGGTACCCCAAATGGTCAGGCT
T20_Lep_R	GCATACTAGTGCCATCGCCGCCGGC
T20_Lep_F	GCATGGTACCGAAGTAGATGCAGTA
T22_Lep_R	GCATACTAGTGCCCTGTGGATCGGC
T22_Lep_F	GCATGGTACCGGTCTCGAAGACGAC
HHV8_G158L_Lep_F	AGTAGAATGACAGCGCTATTGTTAAGCATTGCATTATTGGCCAC
HHV8_G158L_Lep_R	GTGGCCAATAATGCAATGCTTAACAATAGCGCTGTCATTCTACT
T7 ampli TNT Lep F	ATAGTATAATACGACTCACTATAGGAAACCACCATGGCGAATTCCACC
ampli Lep TNT end R	CTATTAATGGATGCCGCC
Bak_BIFC_F1 annealing	GGCCGCATCCTGAACGTGCTGGTGGTTCTGGGTGTGGTTCTGTTGGGCCAG
Bak_BIFC_F2 annealing	TTTGTGGTACGAAGATTCTTCAAATCATGAGC
Bak_BIFC_R1 annealing	AACCACACCCAGAACCACCAGCACGTTCAGGATGC
Bak_BIFC_R2 annealing	GGCCGCTCATGATTTGAAGAATCTTCGTACCACAAACTGGCCCAACAG
Bax_BIFC_F1 annealing	GGCCGCACGTGGCAGACCGTGACCATCTTTGTGGCGGGAGTGCTCACC
Bax_BIFC_F2 annealing	GCCTCGCTCACCATCTGGAAGAAGATGGGCTGAGC
Bax_BIFC_R1 annealing	CGCCACAAAGATGGTCACGGTCTGCCACGTGC
Bax_BIFC_R2 annealing	GGCCGCTCAGCCCATCTTCTTCCAGATGGTGAGCGAGGCGGTGAGCACTCC
T20_VN_in-fusion_F	GCGGGAGTAGCGGCCGCGCCATCGCCGCCGGCGTG
T20_VN/VC_in-fusion_R	TGGATCCCCGCGGCCGCTCAGAAGTAGATGCAGTA
T20_VC_in-fusion_F	AGCAGAAGAGCGGCCGCGCCATCGCCGCCGGCGTG
T22_VN_in-fusion_F	GCGGGAGTAGCGGCCGCGCCCTGTGGATCGGC
T22_VN/VC_in-fusion_R	TGGATCCCCGCGGCCGCTCAGGTCTCGAAGACGAC
T22_VC_in-fusion_F	AGCAGAAGAGCGGCCGCGCCCTGTGGATCGGC
HHV8_FL_BIFC_F	AAGGAAAAAAGCGGCCGCGACGAGGACGTTTTGCC
HHV8_DTM_BIFC_R	TTTTCCTTTTGCGGCCGCTCATCCCCTTCTTCTGGTAAG
HHV8_FL_BIFC_ R	TTTTCCTTTTGCGGCCGCTCATCTCCTGCTCATCGCG
HHV8_T20_BIFC_PCR1 R1	CACACGCCGGCGGCGATGGCTTCTCCGTCCCCTTC
HHV8_T20_BIFC_PCR2 R2	GCAGTAGCCGATGAACAGGGCGCCGCACACGCCGG
HHV8_T20_BIFC_PCR3 R3	TTGCGGCCGCTCAGAAGTAGATGCAGTAGCCGATG
HHV8_T20_BIFC_PCR4 R4	TTTTCCTTTTGCGGCCGCTCA
Bcl2_FL_BIFC_F	AAGGAAAAAAGCGGCCGC ATGGCGCACGCTGGGAG
Bcl2_FL_BIFC_R	TTTTCCTTTTGCGGCCGCTCACTTGTGGCTCAGAT
Bcl2_DTM_BIFC_R	TTTTCCTTTTGCGGCCGCTCAAGGAGAAATCAAACAGAG
Bcl2 G227L_R	CAGTTTGGCCCTGGTGCTAGCTTGCATCAC
Bcl2 G227L_F	CAGGGTGATGCAAGCTAGCACCAGGGCCAA
MyxV_G158I_BIFC_R	GCGATACCATTTCAGAATGATATACGCCACAAAGCCCACC
MyxV_G158I_BIFC_F	GGTGGGCTTTGTGGCGTATATCATTCTGAAATGGTATCGC
MyxV_G158I_pVOTE_F	GGGCTTCGTGGCCTACATCATCCTGAAGTGGTAC
MyxV_G158I_pVOTE_R	GTACCACTTCAGGATGATGTAGGCCACGAAGCCC
MyxV A82F_R	GCAGGGTCAGGGTGAACAGCTTCACGCTGG
MyxV A82F_F	CCAGCGTGAAGCTGTTCACCCTGACCCTGC
MyxV A71F_R	GCTGGCGTCTCTCAGGAAGAAGGTCAGGGTGTTC
MyxV A71F_F	GAACACCCTGACCTTCTTCCTGAGAGACGCCAGC

Name	sequence
Bax_G179I_BlaTM_R	GCTCGCGGTCAGCACGATCGCCACAAAAATGGTC
Bax_G179I_BlaTM_F	GACCATTTTTGTGGCGATCGTGCTGACCGCGAGC
Bax_A183I_BlaTM_R	TCCAAATGGTCAGGCTTATGGTCAGCACGCCCGCC
Bax_A183I_BlaTM_F	GGCGGGCGTGCTGACCATAAGCCTGACCATTTGGA
Bax_G179I/A183I_BlaTM_F	GGCGATCGTGCTGACCATAAGCCTGACCATTTGGA
Bax_G179I/A183I_BlaTM_R	TCCAAATGGTCAGGCTTATGGTCAGCACGATCGCC
Bax_FL_G179I_F	CGAGGCGGTGAGCACTATCGCCACAAAGATGGTC
Bax_FL_G179I_R	GACCATCTTTGTGGCGATAGTGCTCACCGCCTCG
Bax_FL_A183I_F	CCAGATGGTGAGCGAGATGGTGAGCACTATCGCC
Bax_FL_A183I_R	GGCGATAGTGCTCACCATCTCGCTCACCATCTGG
VC-BoHV+3_BIFC_F	GGCCGCCCAAACTTGAAGCTTCCTAGCCCCCGCTCCAGATG
VC-BoHV+3_BIFC_R	CATCTGGAGCGGGGGCTAGGAAGCTTCAAGTTTGGGCGGCC
VC-BoHV+2_BIFC_F	GGCCGCCCAAACTTGAAGCTTAGCCCCCGCTCCAGATG
VC-BoHV+2_BIFC_R	CATCTGGAGCGGGGGCTAAGCTTCAAGTTTGGGCGGCC
VC-BoHV+1_BIFC_F	GGCCGCCCAAACTTGAAGCTTCCCCGCTCCAGATG
VC-BoHV+1_BIFC_R	CATCTGGAGCGGGGAAGCTTCAAGTTTGGGCGGCC
KpnI-cMyc-HHV8_pCAGGS_F	GCATGGTACCATGGAGCAGAAGCTGATCAGCGAGGAGGACCTGGACGAGGACGTTTTGCCTGG
XhoI-HHV8 FL_pCAGGS_R	TGCTAGCTCGAGTTATCTCCTGCTCATCGC
XhoI-HHV8 DTM_pCAGGS_F	TGCTAGCTCGAGTTATCCCCTTCTTCTGGTAAG
HHV8_T20_pCAGGS_PCR1 R1	ACGCCGGCGGCGATGGCTCCCCTTCTTCTGGTAAG
HHV8_T20_pCAGGS_PCR2 R2	GTAGCCGATGAACAGGGCGCCGCACACGCCGGCGG
HHV8_T20_pCAGGS_PCR3 R3	ACGTCTCGAGTCAGAAGTAGATGCAGTAGCCGATG
In-fusion _pCAGGS _T20_R	GATCTGCTAGCTCGATCAGAAGTAGATGCAGTAGCCG
KpnI-cMyc-Bcl2_pCAGGS_F	GCATGGTACCATGGAGCAGAAGCTGATCAGCGAGGAGGACCTGGCGCACGCTGGGAGAAC
XhoI-Bcl2 FL_pCAGGS_R	TGCTAGCTCGAGTCACTTGTGGCTCAG
XhoI-Bcl2 DTM_pCAGGS_R	TGCTAGCTCGAGTCAGGAGAAATCAAACAG
Bcl2_T20_pCAGGS_R1	ACGCCGGCGGCGATGGCGGAGAAATCAAACAGAGG
cMyc-MyxV_pCAGGS_F	ACCATGGAGCAGAAGCTGATCAGCGAGGAGGACCTGAGCAGACTGAAGACC
MyxV-T20 _pCAGGS_PCR1 R	ACGCCGGCGGCGATGGCCTTGCAGCCGCCGCTTCT
MyxV-FL_pCAGGS R	GATCTGCTAGCTCGATCAGGTGCCTCTGTACCACT
MyxV-DTM-pCAGGS_R	TCACTTGCAGCCGCCGC
T20_pCAGGS_IF_R(new)	CTCGAGCATGCCCGGTCAGAAATAAATGCAATA
VAcV_FL_infusion_R	GATCTGCTAGCTCGAGATCATGTACTTCAGGGTCTTG
InFusion VacV FL reverse	GATCTGCTAGCTCGACTAGCTCGAGATCATGTACTTCAGG
InFusion VacV DTM reverse	GATCTGCTAGCTCGACTAGGTGGAGGTGTTGGGG
pVote cmyc to pCAGGSKpnI_F	GCTCATCGATGCATGCCACCATGGAGCAGAAGCTGATCAGC
pVote to pCAGGS KpnI_R	CTCGAGCATGCCCGGATTCCCGGGAGCTCGAGC
MyxV_G158I_IF_pCAGGS_R1	CACTTCAGGATGATGTAGGCCACGAAGCCCACCAC
MyxV_G158I_IF_pCAGGS_R2	CTCGAGCATGCCCGGTCAGGTGCCTCTGTACCACTTCAGGATGAT
cMyc_NdeI_pVOTE2_F	AACACGATAATCATAATGGAGCAGAAGCTGATCAGCG
Bcl2_FL_NdeI_pVOTE2_R	GGGAGCTCGAGCATATCACTTGTGGCTCAGATAGGC
Bcl2_DTM_NdeI_pVOTE2_R	GGGAGCTCGAGCATATCAGGAGAAATCAAACAGAGGCCG
T20_NdeI_pVOTE2_R	GGGAGCTCGAGCATATCAGAAGTAGATGCAGTAGCCG
T22_NdeI_pVOTE2_R	GGGAGCTCGAGCATATCAGGTCTCGAAGACGACGG
MyxV_FL_NdeI_pVOTE2_R	GGGAGCTCGAGCATATCAGGTGCCTCTGTACCACTTCAGG
MyxV_DTM_NdeI_pVOTE2_R	GGGAGCTCGAGCATATCACTTGCAGCCGCCGCTTCTCACG
HHV8_FL_NdeI_pVOTE2_R	GGGAGCTCGAGCATATTATCTCCTGCTCATCGCGACC
HHV8_DTM_NdeI_pVOTE2_R	GGGAGCTCGAGCATATTATCCCCTTCTTCTGGTAAGCACC
VacV_FL_NdeI_pVOTE2_R	GGGAGCTCGAGCATAGATCATGTACTTCAGGGTCTTGT
VacV_DTM_NdeI_pVOTE2_R	GGGAGCTCGAGCATAGGTGGAGGTGTTGGGGATTG
HHV4_anneal_BlaTM_F1	CTAGCTTTAGCTGGACCCTGTTTCTGGCGGGCCTG
HHV4_anneal_BlaTM_F2	ACCCTGAGCCTGCTGGTGATTTGCAGCTATCTGTTTATTGG
HHV4_anneal_BlaTM_R1	GATCCCAATAAACAGATAGCTGCAAATCACCAGCA
HHV4_anneal_BlaTM_R2	GGCTCAGGGTCAGGCCCGCCAGAAACAGGGTCCAGCTAAAG
BoHV_anneal_BlaTM_F1	CTAGCTGGCTGTTTCCGATGTTTGCGATTAGCGGC
BoHV_anneal_BlaTM_F2	CTGGTGCTGACCGTGGGCGTGGCGCGCGG
BoHV_anneal_BlaTM_R1	GATCCCGCGCGCCACGCCCACGGTCAGCACCAGGC
BoHV_anneal_BlaTM_R2	CGCTAATCGCAAACATCGGAAACAGCCAG
VacV_anneal_BlaTM_F1	CTAGCGAATATCTGAAACTGATTGGCATTACCGCG
VacV_anneal_BlaTM_F2	ATTATGTTTGCGACCTATAAAACCCTGGG
VacV_anneal_BlaTM_R1	GATCCCCAGGGTTTTATAGGTCGCAAACATAATCG
VacV_anneal_BlaTM_R2	CGGTAATGCCAATCAGTTTCAGATATTCG
OrfV_anneal_BlaTM_F1	CTAGCGCGCTGGGCGTGGGCGCGGTGGTGGCGGGC
OrfV_anneal_BlaTM_F2	GTGGGCATGCTGCTGCTGGGCGTGCGCGTGCTGCGCGG

Name	sequence
OrfV_anneal_BlaTM_R1	GATCCCGCGCAGCACGCGCACGCCCAGCAGCAGCA
OrfV_anneal_BlaTM_R2	TGCCCACGCCCGCCACCACCGCGCCCACGCCCAGCGCG
T20_anneal_BlaTM_F1	CTAGCGCGATTGCGGCGGGCGTGTGCGGCGCGCTG
T20_anneal_BlaTM_F2	TTTATTGGCTATTGCATTTATTTTGG
T20_anneal_BlaTM_R1	GATCCCAAAATAAATGCAATAGCCAATAAACAGCGC
T20_anneal_BlaTM_R2	GCCGCACACGCCCGCCGCAATCGCG
HHV4_BlaTM_InFusion_F	TGCTAATCGAGCTAGCTTTAGCTGGA
HHV4_BlaTM_InFusion_R	GCCAGTTTGTGGATCCCGATAAATAAA
BoHV_BlaTM_InFusion_F	TGCTAATCGAGCTAGCTGGTTATTTCCCA
BoHV_BlaTM_InFusion_R	GCCAGTTTGTGGATCCCTCTCGCCACACCCA
VacV_BlaTM_InFusion_F	TGCTAATCGAGCTAGCGAGTACCTGAAG
VacV_BlaTM_InFusion_R	GCCAGTTTGTGGATCCCCAGGGTCTTGT
T20_BlaTM_InFusion_F	TGCTAATCGAGCTAGCGCCATCGCCGCCG
T20_BlaTM_InFusion_R	GCCAGTTTGTGGATCCCGAAGTAGATGC
OrfV_BlaTM_InFusion_F	TGCTAATCGAGCTAGCGCCCTGGGCGTG
OrfV_BlaTM_InFusion_R	GCCAGTTTGTGGATCCCGCGCAGCACGC
Seq BIFC CMV-promoter	CGCAAATGGGCGGTAGGCGTG
Seq BIFC Ct Reverse	CAGCCAGCCGCGGCC
Seq pCAGGS F	CCTTCTTCTTTTTCCTACAGC
Seq pCAGGS R	GATGTCCCCATAATTTTTGGCAGAGGG
Seq pVECTOR2 F	GGCCCCCCGAACCACGGGGAC
Seq BlaTM_R	CTGAACACCATAGGTCAGGGTGGTAACCAGG
Seq_N_BlaTM_F	TTATCTACACGACGGGGAGTCAGGCAACTATG
Seq_C_BlaTM_F	GAAGCCATACCAAACGACGAGCGTGACACC
