# Supplementary material for: Viral Bcl2s’ transmembrane domain interact with host Bcl2 proteins to control cellular apoptosis
Source: Nat Commun. 2020 Nov 27;11:6056. doi: 10.1038/s41467-020-19881-9 (PMC7695858; doi:10.1038/s41467-020-19881-9)
Supplement: Supplementary file 3 — Reporting Summary [file 41467_2020_19881_MOESM3_ESM.pdf]

Corresponding author(s): Luis Martinez-Gil

Last updated by author(s): Oct 14, 2020

Reporting Summary

Nature Research wishes to improve the reproducibility of the work that we publish. This form provides structure for consistency and transparency in reporting. For further information on Nature Research policies, see our Editorial Policies and the Editorial Policy Checklist.

Statistics

For all statistical analyses, confirm that the following items are present in the figure legend, table legend, main text, or Methods section.

n/a	Confirmed	
[ ]	[x]	The exact sample size (*n*) for each experimental group/condition, given as a discrete number and unit of measurement
[ ]	[x]	A statement on whether measurements were taken from distinct samples or whether the same sample was measured repeatedly
[ ]	[x]	The statistical test(s) used AND whether they are one- or two-sided<br>*Only common tests should be described solely by name; describe more complex techniques in the Methods section.*
[x]	[ ]	A description of all covariates tested
[ ]	[x]	A description of any assumptions or corrections, such as tests of normality and adjustment for multiple comparisons
[ ]	[x]	A full description of the statistical parameters including central tendency (e.g. means) or other basic estimates (e.g. regression coefficient) AND variation (e.g. standard deviation) or associated estimates of uncertainty (e.g. confidence intervals)
[ ]	[x]	For null hypothesis testing, the test statistic (e.g. *F*, *t*, *r*) with confidence intervals, effect sizes, degrees of freedom and *P* value noted<br>*Give P values as exact values whenever suitable.*
[x]	[ ]	For Bayesian analysis, information on the choice of priors and Markov chain Monte Carlo settings
[x]	[ ]	For hierarchical and complex designs, identification of the appropriate level for tests and full reporting of outcomes
[x]	[ ]	Estimates of effect sizes (e.g. Cohen's *d*, Pearson's *r*), indicating how they were calculated

Our web collection on statistics for biologists contains articles on many of the points above.

Software and code

Policy information about availability of computer code

Data collection	Olympus FV10-ASV v04.02.01.20 , BD FACSDiva
Data analysis	ΔG prediction server v1.0,TMHMM Server v. 2.0, Preddimer, Clustal Omega (EMBL-EBI site), Gene Expression Similarity Investigation Suite (Genesis), Microsoft Office, Fiji-Image J, Image Reader 8.1 ,GraphPad Prisma v.8

For manuscripts utilizing custom algorithms or software that are central to the research but not yet described in published literature, software must be made available to editors and reviewers. We strongly encourage code deposition in a community repository (e.g. GitHub). See the Nature Research guidelines for submitting code & software for further information.

Data

Policy information about availability of data

All manuscripts must include a data availability statement. This statement should provide the following information, where applicable:

- Accession codes, unique identifiers, or web links for publicly available datasets
- A list of figures that have associated raw data
- A description of any restrictions on data availability

The data that support the findings of this study are available from the corresponding author upon reasonable request. Source data are provided with this paper.

Field-specific reporting

Please select the one below that is the best fit for your research. If you are not sure, read the appropriate sections before making your selection.

☒ Life sciences ☐ Behavioural & social sciences ☐ Ecological, evolutionary & environmental sciences

For a reference copy of the document with all sections, see nature.com/documents/nr-reporting-summary-flat.pdf

Life sciences study design

All studies must disclose on these points even when the disclosure is negative.

Sample size	The sample size was determined experimentally base on the variability of the results. n≥3
Data exclusions	No data was excluded
Replication	All attempts at replication were successful. Replicates were performed independently and experimental findings were reproducible. n≥3
Randomization	The nature of the experiments in our work do not require randomization.
Blinding	When possible samples were assigned a code number. Identification of the samples and measuring were one by different persons.

Reporting for specific materials, systems and methods

We require information from authors about some types of materials, experimental systems and methods used in many studies. Here, indicate whether each material, system or method listed is relevant to your study. If you are not sure if a list item applies to your research, read the appropriate section before selecting a response.

Materials & experimental systems

n/a	Involved in the study	
☐	☒	Antibodies
☐	☒	Eukaryotic cell lines
☒	☐	Palaeontology and archaeology
☒	☐	Animals and other organisms
☒	☐	Human research participants
☒	☐	Clinical data
☒	☐	Dual use research of concern

Methods

n/a	Involved in the study	
☒	☐	ChIP-seq
☐	☒	Flow cytometry
☒	☐	MRI-based neuroimaging

Antibodies

Antibodies used	Antibodies used in this study were β-actin (Santa Cruz Biotechnology SC-47778), c-Myc (Sigma PLA0001 or Roche 11667149001), Histone 3 (Sigma H0164) and Flag (Sigma B3111). Additionally a goat anti-mouse IgG-peroxidase conjugate (Sigma DC02L) was used as a secondary. An Alexa Fluor 488 anti rabbit (Life Technologies A21206) and a rabbit anti c-Myc antibody (Sigma PLA0001) was used to obtain the fluorescence micrographies. In Western blot assays the antibodies were diluted 1:10000 in TBS-T and for fluorescence micrographies were diluted 1:1000 in TBS-T
Validation	We only used commercially avalaible antibodies. No validation was done in our laboratory beyond the use of a negative control.

Eukaryotic cell lines

Policy information about cell lines

Cell line source(s)	HEK293T, HCT119 and Hela Cells were obtained from ATCC through the tissue culture and flow cytometry core facilities of the SCSIE (University of Valencia).
Authentication	No autentification of the cell lines was performed.
Mycoplasma contamination	Cells were tested for Mycolasma (and resulted negative) by the tissue culture and flow cytometry core facilities of the SCSIE (University of Valencia)
Commonly misidentified lines (See ICLAC register)	No commonly misidentified lines were used in this study.

Flow Cytometry

Plots

Confirm that:

- [x] The axis labels state the marker and fluorochrome used (e.g. CD4-FITC).
- [x] The axis scales are clearly visible. Include numbers along axes only for bottom left plot of group (a 'group' is an analysis of identical markers).
- [ ] All plots are contour plots with outliers or pseudocolor plots.
- [x] A numerical value for number of cells or percentage (with statistics) is provided.

Methodology

Sample preparation	Preparation for HeLa cells was done according to the specifications of BD Annexin V; FITC Apoptosis Detection Kit II
Instrument	BD LSR-Fortessa (Beckton Dikinson)
Software	All flow cytometry countings were done at the tissue culture and flow cytometry core facilities of the SCSIE (university of Valencia, www.uv.es)
Cell population abundance	The cell populations was determined by the flow cytometry core facilites (SCSIE, University of Valencia) personnel following the specifications of the BS Annexin V; FITC Apoptosis Detection Kit II
Gating strategy	The gating was impose by the flow cytometry core facilites (SCSIE, University of Valencia) personnel following the specifications of the BS Annexin V; FITC Apoptosis Detection Kit II

- [x] Tick this box to confirm that a figure exemplifying the gating strategy is provided in the Supplementary Information.
